# The Antibacterial Potential of Honeydew Honey Produced by Stingless Bee (*Heterotrigona itama*) against Antibiotic Resistant Bacteria

**DOI:** 10.3390/antibiotics9120871

**Published:** 2020-12-05

**Authors:** Wen-Jie Ng, Nam-Weng Sit, Peter Aun-Chuan Ooi, Kah-Yaw Ee, Tuck-Meng Lim

**Affiliations:** Faculty of Science, Universiti Tunku Abdul Rahman, Kampar 31900, Malaysia; sitnw@utar.edu.my (N.-W.S.); peteracooi@gmail.com (P.A.-C.O.); eeky@utar.edu.my (K.-Y.E.); ltmeng@utar.edu.my (T.-M.L.)

**Keywords:** blossom honey, honeydew, *Apis cerana*, *Geniotrigona*, *Heterotrigona*, agar-well diffusion, bactericidal, endotoxin, synergistic, antibiotic resistant

## Abstract

Scientific studies about the antibacterial effects of honeydew honey produced by the stingless bee are very limited. In this study, the antibacterial activities of 46 blossom and honeydew honeys produced by both honey bees and stingless bees were evaluated and compared. All bacterial isolates showed varying degrees of susceptibility to blossom and honeydew honeys produced by the honey bee (*Apis cerana*) and stingless bee (*Heterotrigona itama* and *Geniotrigona thoracica*) in agar-well diffusion. All stingless bee honeys managed to inhibit all the isolates but only four out of 23 honey bee honeys achieved that. In comparison with *Staphylococcus aureus*, *Escherichia coli* was found to be more susceptible to the antibacterial effects of honey. Bactericidal effects of stingless bee honeys on *E. coli* were determined with the measurement of endotoxins released due to cell lysis. Based on the outcomes, the greatest antibacterial effects were observed in honeydew honey produced by *H. itama*. Scanning electron microscopic images revealed the morphological alteration and destruction of *E. coli* due to the action of this honey. The combination of this honey with antibiotics showed synergistic inhibitory effects on *E. coli* clinical isolates. This study revealed that honeydew honey produced by *H. itama* stingless bee has promising antibacterial activity against pathogenic bacteria, including antibiotic resistant strains.

## 1. Introduction

The antibacterial activity of honey has been known since the 19th century [1]. Recently, the potent inhibitory activity of stingless bee honey has further increased the interest in the application of honey to eradicate antibiotic-resistant bacteria. Studies have shown that stingless bee honeys displayed greater and broader spectrum inhibitory activities than honey produced by Apis honey bees, against Gram-positive *Staphylococcus aureus*, *Staphylococcus epidermidis*, *Streptococcus pyogenes*, *Streptococcus pneumoniae*, *Enterococcus faecalis*, and Gram-negative *Escherichia coli*, *Salmonella* serotype Typhimurium, *Pseudomonas aeruginosa,* and *Klebsiella pneumoniae*, including the multidrug-resistant strains [2,3,4].

Among at least 32 stingless bee species that have been identified in Malaysia, the most abundant species found in meliponiculture are *Heterotrigona itama* and *Geniotrigona thoracica* because they produce higher volume of honey as compared to the other stingless bee species [5,6]. Similar to the honey bees, the stingless bees collect nectar for honey production, but they store the nectar in honey pots instead of hexagonal honeycomb. The honey pots are made of cerumen, which is a mixture that is similar to propolis but with the addition of the mandibular secretion of the stingless bee during its construction [7,8]. The good quality of stingless bee honey is ascribed to the infiltration of phytochemicals from the cerumen pots [9].

Other than the bee types, honey also can be classified based on its botanical sources which are blossom honey and honeydew honey. Blossom honey, also known as floral honey, is the most common type of honey worldwide, whereas honeydew honey is originated from the secretions of living parts of plants or excretions of plant-sucking insects on the plants [10]. Although scientific studies on honeydew honey are rather more limited than those on blossom honey, the antibacterial effects of honeydew honey have been proven in several studies, even more potent than the blossom honey [11,12,13,14]. In general, not all types of honey exhibit similar level of antibacterial activity since it is greatly associated with the bee species, nectar origins, and other intrinsic factors like osmotic effect, acidity, hydrogen peroxide, and phytochemicals [15,16].

Until now, no studies have been conducted on the antibacterial effects of honeydew honey produced by stingless bees, specifically *H. itama* and *G. thoracica*. Therefore, the main objective of this study was to evaluate the in vitro antibacterial activity of these honey types against selected pathogenic bacteria including both antibiotic sensitive and resistant strains. To date, only a few honey types have been approved for medicinal use to treat infections, which are manuka honey and Revamil honey [17]. Hence, the outcomes of this study could provide useful information on the potential of Malaysian stingless bee honeydew honey as an antibacterial agent in the health care sector.

## 2. Results

### 2.1. Inhibitory Effects

The inhibitory outcomes of 46 tested honey samples are tabulated in Table 1. According to the results shown, 21 honey bee honey samples were able to inhibit *E. coli* but only six honey bee honey samples managed to inhibit *S. aureus*. In comparison, all 23 stingless bee honey samples were able to inhibit the growth of *E. coli* as well as *S. aureus*. Generally, the stingless bee honey was suggested to have a stronger antibacterial effect compared with the honey bee honey, with the zone of inhibition spanning 0.7 cm to 1.7 cm and 0 cm to 1.1 cm, respectively.

### 2.2. Bactericidal Effects

The bactericidal effect of stingless bee honey on *E. coli* which was assessed with endotoxin assay is tabulated in Table 2. Endotoxin was measured in endotoxin units per milliliter (EU/mL). One EU equals approximately to 0.1 to 0.2 ng endotoxin/mL of solution. The release of endotoxin due to bacterial destruction was shown to be increased significantly from 0-h to 24-h of incubation. The highest levels of endotoxin were detected in *E. coli* ATCC 25922 after treated with *H. itama* honeydew honey (S1–S8) with 1.56 EU/mL to 1.97 EU/mL at 0-h then 2.21 EU/mL to 2.38 EU/mL at 24-h. For *E. coli* ATCC 35218, the highest endotoxin levels, 1.51 EU/mL to 1.88 EU/mL were detected at 0-h then 2.20 EU/mL to 2.32 EU/mL at 24-h after treated with the same honey type (S1–S8).

Next, the blossom honey produced by *G. thoracica* (S17–S23) was able to induce the release of endotoxin from 1.50 EU/mL to 1.68 EU/mL at 0-h and 2.15 EU/mL to 2.31 EU/mL at 24-h in *E. coli* ATCC 25922; 1.48 EU/mL to 1.58 EU/mL at 0-h and 2.145 EU/mL to 2.29 EU/mL at 24-h in *E. coli* ATCC 35218. In contrast, the *H. itama* blossom honey (S9–S16) displayed the lowest endotoxin levels, with 1.27 EU/mL to 1.38 EU/mL at 0-h and 2.00 EU/mL to 2.15 EU/mL at 24-h in *E. coli* ATCC 25922; 1.25 EU/mL to 1.38 EU/mL at 0-h and 2.00 EU/mL to 2.12 EU/mL at 24-h in *E. coli* ATCC 35218.

**Figure 1:**
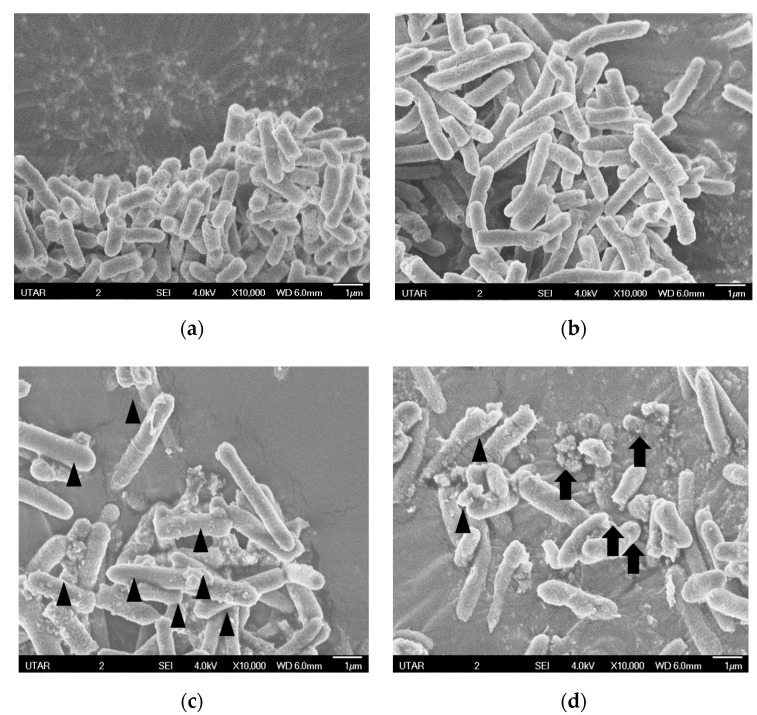
SEM images of the antibacterial effect of *H. itama* honeydew honey against *E. coli*: (**a**) Negative control *E. coli* ATCC 25922 (without honey); (**b**) Negative control *E. coli* ATCC 35218 (without honey); (**c**) *E. coli* ATCC 25922 treated with stingless bee honeydew honey, showing that the cells formed blebs with rough surface (arrowhead); (**d**) *E. coli* ATCC 35218 treated with stingless bee honeydew honey, showing ruptured and lysed cells (arrow).

The impacts of *H. itama* blossom honey on the morphology of *E. coli* were observed at 10,000 magnification power using a scanning electron microscope (SEM). The normal morphology and intact structures of *E. coli* ATCC 25922 and ATCC 35218 are shown in Figure 1a,b, respectively. Figure 1c shows the formation of blebs on the rough surfaces of *E. coli* ATCC 25922 after treated with honeydew honey. Ruptured and lysed *E. coli* ATCC 35218 due to the action of honeydew honey can be seen in Figure 1d.

### 2.3. Antibacterial Factors

Table 3 shows sugar solution, hydrogen peroxide solution, acid solution and gallic acid solution samples formulated based on the physicochemical properties of *H. itama* blossom honey displayed no inhibitory and minimal bactericidal effects on *E. coli*.

### 2.4. Interactive Effects with Antibiotics

Together with *E. coli* (ATCC 25922 and 35218), the combined inhibitory effects of *H. itama* honeydew honey and antibiotics were tested on four clinical isolates of *E. coli*. The antibiotic susceptibility profile of each clinical strain is presented in Table 4. Other than *E. coli* 1 which was resistant to ampicillin, other isolates were resistant to at least two antibiotics especially *E. coli* 3 which was resistant to three out of four tested antibiotics. As displayed in Table 5, a combination of honey and antibiotic was considered synergistic when the scored zone of inhibition for the combination was bigger than the zone of inhibition of honey and antibiotic separately. The results revealed that the addition of honeydew honey showed synergistic antibacterial effect with ampicillin with larger diameter of inhibition zones against *E. coli* 1, from 0.7 ± 0.1 cm for honeydew honey alone and no zone of inhibition for ampicillin alone to 0.9 ± 0 cm for the combination; *E. coli* 2, from 0.7 ± 0 cm for honeydew honey alone and 0.7 ± 0.1 cm for ampicillin alone to 1.3 ± 0 cm for the combination; *E. coli* 3, from 1.0 ± 0.1 cm for honeydew honey alone and no zone of inhibition for ampicillin alone to 1.4 ± 0 cm for the combination; *E. coli* ATCC 25922, from 1.2 ± 0 cm for honeydew honey alone and 1.0 ± 0.1 cm for ampicillin alone to 1.7 ± 0 cm for the combination.

Similar results were recorded for the combination of *H. itama* honeydew honey with gentamicin, with zone of inhibition of 1.3 ± 0.1 cm for the combination when compared with honeydew honey and gentamicin alone, with zone of inhibition of 0.7 ± 0 cm and 1.0 ± 0 cm, respectively against *E. coli* 2; zone of inhibition of 1.4 ± 0 cm for the combination while honeydew honey and gentamicin alone, with zone of inhibition of 1.0 ± 0.1 cm and no zone of inhibition, respectively against *E. coli* 3; zone of inhibition of 2.2 ± 0 cm for the combination while honeydew honey and gentamicin alone, with zone of inhibition of 1.2 ± 0 cm and 2.0 ± 0 cm, respectively, against *E. coli* ATCC 25922.

## 3. Discussion

### 3.1. Inhibitory and Bactericidal Effects

The antibacterial effects of honeybee and stingless bee honey samples were tested against two types of bacteria, Gram-positive *S. aureus* and Gram-negative *E. coli* which are the two most common bacterial nosocomial infections [18]. According to Table 1, all stingless bee honeys were found to exhibit inhibitory effects against *S. aureus* and *E. coli*. The diameters of inhibition zones exerted by stingless bee honey on *S. aureus* (ATCC 25923) and *E. coli* (ATCC 25922 and ATCC 35218) were significantly larger than those of honey bee honey. Thus, stingless bee honey was claimed to possess greater antibacterial effects than honey bee honey.

Comparative investigation of antibacterial effects between stingless bee and honey bee honey samples is very limited. Stingless bee honey had greater antibacterial effects than two honey bee honeys with broader antibacterial spectrum and larger zone of inhibition on *S. aureus* (1.8 ± 0 cm), *E. coli* (1.6 ± 0 cm), *Proteus vulgaris* (2.4 ± 0 cm), *Shigella sonnei* (1.2 ± 0 cm), and *Klebsiella* sp. (8.2 ± 0.5 cm) [18]. However, one of the honey bee honeys was found to exhibit larger zone of inhibition on *E. coli* (2.6 ± 0 cm), *P. vulgaris* (2.6 ± 0 cm), *S. sonnei* (1.8 ± 0 cm), *Salmonella paratyphi* (1.4 ± 0 cm), and *Klebsiella* sp. (1.7 ± 0 cm), but unable to inhibit *S. aureus* [18]. Another study also stated stingless bee honey exhibited the highest mean inhibition (2.2 ± 0.4 cm) compared to other honey bee honeys (2.1 ± 0.3 cm and 1.8 ± 0.2 cm) on all the tested bacteria, including *S. aureus*, *E. coli,* and resistant clinical isolates *S. aureus*, *E. coli,* and *K. pneumoniae* [3].

As reported in several studies, *S. aureus* was found to be more susceptible to the inhibitory action of stingless bee honey than *E. coli* [3,19,20,21,22]. However, in this study, *E. coli* was found to be more sensitive to the antibacterial action of stingless bee honey. These results were in agreement with other studies that showed honey exhibited greater inhibitory effect on *E. coli* with larger inhibition zone and lower MIC than *S. aureus* [23,24]. Methanol, ethanol, and ethyl acetate extracts of raw and processed honey were found to be more effective to Gram-negative *E. coli*, *P. aeruginosa,* and *Salmonella typhi* than Gram-positive *S. aureus*, *B. cereus*, *Bacillus subtilis,* and *Micrococcus luteus* [25]. The inhibition zones exerted on Gram-negatives (1.3–3.8 cm) were significantly larger than Gram-positives (0.7–2.4 cm). Honey was found to target the cell wall and lipopolysaccharide outer membrane of *E. coli*, causing the cell wall destruction and increased permeability of the outer membrane, eventually leading to cell lysis [26].

Although honeydew honey is increasingly valued due to its pronounced antibacterial potential, there is no scientific report about the antibacterial effects of stingless bee honeydew honey [14]. The antibacterial effects of stingless bee honey that are reported in the scientific articles only focusing on the blossom type. Hence, in Table 1, the honey samples were further categorized into honeydew and blossom types. Larger inhibition zones were found in *E. coli* ATCC 25922 and ATCC 35218 due to the inhibitory effect of honeydew honey produced by *H. itama* with diameters of 1.3 ± 0.3 cm and 1.3 ± 0.2 cm, respectively comparing with the inhibition zones exerted by other stingless bee blossom honeys. By using the supporting evidence of honeydew honey produced by honey bees, Slovakian honey was found to have exceptional antibacterial activity against multi-drug resistant *Stenotrophomonas maltophilia* isolated from cancer patients and it was more efficient than Manuka honey [12]. Italian honeydew honey was also found to have higher bacteriostatic and bactericidal activities on *S. aureus*, *E. faecalis*, *E. coli*, *Proteus mirabilis*, and *P. aeruginosa* than other blossom honeys [13]. A study demonstrated that honeydew honey-based membranes had strong antibacterial activities against multidrug-resistant strains of *E. coli*, *P. aeruginosa*, *P. mirabilis*, and *Staphylococcus pseudointermedius* which were isolated from canine wound infections [27].

Although agar-well diffusion has been the most commonly used method to evaluate the antibacterial effects of honey, this method does not differentiate between inhibitory and bactericidal activity as well as to allow quantification of bactericidal activity or kinetics of killing [1,2]. Thus, an endotoxin assay was carried out to confirm the bactericidal effect of stingless bee honeys against *E. coli*. In Table 2, it can be observed that the treatment of stingless bee honey on *E. coli* led to the release of endotoxin. Honey treatment was able to cause destruction of cell wall and disintegration of lipopolysaccharide outer membrane of *E. coli* with endotoxin release at bactericidal concentration [26]. As a general observation, the level of endotoxin released due to bacterial destruction after treated with honey for 24 h was significantly higher than the 0-h thus indicating more bacteria were killed in longer duration. The outcomes of this study are consistent with a previous study whereby it was reported that incubation with stingless bee honey for 60 min resulted in a significant decrease in the viability of *S. aureus* and *P. aeruginosa* as compared to 0 min of incubation [2]. Based on the outcomes of endotoxin assay, it is confirmed that honeydew honey produced by *H. itama* has greater antibacterial effect specifically bactericidal action, than blossom honeys based on significantly higher level of endotoxin released in both *E. coli* strains.

The antibacterial effect of stingless bee honeydew honey was further verified by scanning electron microscopy; as displayed in Figure 1, *E. coli* cells were observed to suffer loss of structural integrity that caused by the antibacterial mechanisms of action exerted by the honey. *E. coli* treated with honey was reported to possess longer rod and filamentous shapes, indicative of the inhibition of septation and cell division. Furthermore, after longer incubation, spheroplasts, smaller cells, and cell debris were observed in honey-treated *E. coli* samples [26]. Together with the results of endotoxin assay, it is clearly revealed that structural changes in *E. coli* and the damaged cell wall and outer membrane constituted the mechanism underlying the antibacterial effects of stingless bee honeydew honey.

### 3.2. Antibacterial Factors

Hyperosmolality is claimed as one of the antibacterial factors in honey due to its high sugar content, which limits the uptake of water molecules by bacteria for growth. However, as shown in Table 3, the prepared sugar solution (43% fructose, 28% glucose, and 2.0% sucrose, g/100 g) was unable to inhibit both *E. coli* strains. A review mentioned that several studies also had tested the ‘artificial honey’ that was prepared by mixing monosaccharides and disaccharides with the same total sugar content in honey but failed to inhibit any bacterial growth [28]. In an experiment, honey was tested to have 18% of minimum inhibitory concentration in agar-well diffusion method but the same concentration of ‘artificial honey’ was unable to exhibit the same antibacterial effect [29]. Hence, hyperosmolality was considered not to be the predominant antibacterial factor in honey [4,19].

The acidity environment in honey is described to alter the metabolism of bacteria by interfering the enzymatic activities and disrupting plasma membrane integrity [30]. In this study, the acidity of honey was mimicked by using hydrochloric acid solution (pH 3.3) to test against *E. coli* but failed to exhibit any zone of inhibition (Table 3). Although the acidic environment in honey is due to the presence of gluconic acid and has claimed to be one of the antibacterial factors, no significant decrease in antibacterial effect was observed after the acidity of honey was neutralized [31]. Despite the fact that, in this study, stingless bee honey with the lowest pH was found to exhibit more potent antibacterial effect, hence it was suggested that the antibacterial effect of stingless bee honey could be influenced by acidity in combination with other factors [19].

Hydrogen peroxide possesses the ability to cause extensive protein degradation and cellular damage in bacteria, however, the prepared hydrogen peroxide solution (184 µmol/L or µM) in this study was unable to inhibit the growth of *E. coli* (Table 3). A similar outcome was also observed in a study whereby a hydrogen peroxide solution with the concentration of 256.3 μM failed to inhibit *E. coli* [32]. A possible reason of such outcomes could be due to insufficient concentration level of hydrogen peroxide to exhibit antibacterial effect. The concentration of 3% hydrogen peroxide which is commonly used as an antiseptic agent in a laboratory is approximately 880 mM [25]. Thus, the difference between concentration of prepared hydrogen peroxide solutions and 3% hydrogen peroxide was too large, therefore, the strength of hydrogen peroxide in the prepared solution was not sufficient to exhibit any inhibitory effect against bacteria. Although stingless bee honey was still able to inhibit bacterial growth after being treated with catalase, the antibacterial potency was greatly reduced by five-fold [4]. Such data showed the importance of hydrogen peroxide to the antibacterial effect of stingless bee honey, but it is undeniable that there are also other components present in the honey that can inhibit the growth of bacteria. The stingless bee honey was found not to contain methylglyoxal (MGO), dihydroxyacetone or phenolics characteristic of manuka plant nectars. Due to no MGO was detected in stingless bee honey, non-peroxide antibacterial activity of stingless bee honey was postulated to be contributed by other factors, for example phytochemicals [32].

As shown in Table 3, gallic acid solution on (104 mg GAE/kg) which was used to represent phenolic compounds in the honey failed to inhibit *E. coli*. A study explained that the total food extracts may be more beneficial and efficient than isolated constituents, since a bioactive individual compound can change its properties in the presence of other compounds, corresponding to a synergistic antibacterial effect [33]. In this study, honey extract, which consisted of five flavonoids (naringenin, kaempferol, apigenin, pinocembrin and chrysin) and nine phenolic acids (protocatequic acid, *p*-hydroxibenzoic acid, caffeic acid, chlorogenic acid, vanillic acid, *p*-coumaric acid, benzoic acid, ellagic acid, and cinnamic acid), was able to inhibit *S. aureus*, *B. subtilis*, *K. pneumoniae,* and *E. coli*. The study also concluded that the phenolic compounds in honey are partially responsible for the antibacterial activity of honey [33]. The antibacterial mechanisms of phenolic compounds were claimed to destruct bacterial membrane, prevent biofilm formation and inhibit virulence factors including toxins and enzymes, furthermore, phenolic compounds were found to diminish antibiotic resistance of pathogenic bacteria [34].

Each hydrogen peroxide and phenolic compounds were found to be insufficient to exhibit antibacterial effects in honey [14]. However, hydrogen peroxide was said to be able to accelerate the auto-oxidation process of phenolic compounds to generate more reactive oxygen species. Thus, the synergism between hydrogen peroxide and phenolic compounds led to greater DNA damage and inhibited the multiplication of bacterial cells [14]. Still, the exact phenolic compounds to have synergism with hydrogen peroxide have yet to be identified. Therefore, it could be another reason to explain the inability of prepared gallic acid solution to exhibit any inhibitory effects against *E. coli* in the current study. The outcomes suggested that the antibacterial effect of stingless bee honeydew honey is due to the interactive action among different factors instead of depending solely on one of these physicochemical properties.

### 3.3. Interactive Effects with Antibiotics

According to Table 5, *E. coli* isolates, including antibiotic resistant clinical strains, were found to have higher susceptibility to the mixture of stingless bee honeydew honey and antibiotics. Larger inhibition zones exhibited by the combination of honey and ampicillin was considered synergistic when the scored zone of inhibition for the combination was bigger than the zone of inhibition of honey and antibiotic separately [35]. Although not all of the *E. coli* strains tested responded in the same way to these combinational treatments, the honey–ampicillin combination was considered as the most promising, with larger inhibition zones and higher endotoxin levels.

Honey has been tested to work better with beta-lactam antibiotic to inhibit bacteria [36]. An approximate doubling of the inhibition zone was observed due to the action of honey and oxacillin together. In contrast, the combination of honey and gentamicin produced little to no additive effects for all bacteria strains. The combination of honey and gentamicin was also reported had no synergistic effect against methicillin-resistant *S. aureus* (MRSA) [37]. A greater antibacterial effect was also observed in another study whereby the combination of honey and beta-lactam ampicillin displayed larger inhibition zone and higher bactericidal rate. The synergistic effect was believed to cause significant morphological alteration and subsequently bacterial cell lysis [38].

Greater antibacterial effects were believed to be the involvement of honey antibacterial factors and ampicillin together. One of the ways in which a combination of antibacterial compounds works is when both compounds act sequentially, achieving a ‘like plus like’ effect [39]. Hydrogen peroxide which is naturally present in honey can diffuse across the bacterial cell membrane and generate hydroxyl free radicals. Oxidative stress that is caused by the free radicals encourages lipid peroxidation which would disrupt the integrity of cell membrane [40]. Moreover, the formation of hydroxyl free radicals could destruct bacterial DNA [41]. It was believed that the damaged DNA would inhibit the formation of enzyme beta-lactamase which would greatly enhance the susceptibility of bacteria towards the action of ampicillin [38]. On the other hand, gentamicin which is an aminoglycoside that inhibits bacteria by targeting the 30S subunits of the ribosome [42]. Since honey alters the production of protein, including ribosomal proteins in bacteria [43,44], the synergistic effect of honey in combination with gentamicin may be due to these impacts on the protein synthesis pathway, thus inhibiting the growth of bacteria more effectively.

Interestingly, not all *E. coli* strains were found to have higher susceptibility towards the inhibitory effect of the honey–antibiotic combination. It may be due to different responses in these strains toward the stresses induced by the honey and/or antibiotics such as efflux systems or barriers that prevent the entry, accumulation or action of these antibacterial agents [36]. Although the combination of honey and antibiotic may not always work synergistically, honey still can be recommended as a good antibiotic adjuvant. It was stated that antibiotic can act systemically entering from the bottom of the wound bed, while honey acts topically from the top of the wound [36]. The overall effectiveness of stingless bee honeydew honey and antibiotic combinations shown in this study can be suggested as an alternative for wound infection treatment, since *E. coli* is one of the most common bacterial species associated with acute and chronic wound infections [45,46].

## 4. Materials and Methods

### 4.1. Honey Samples

**Figure 2:**
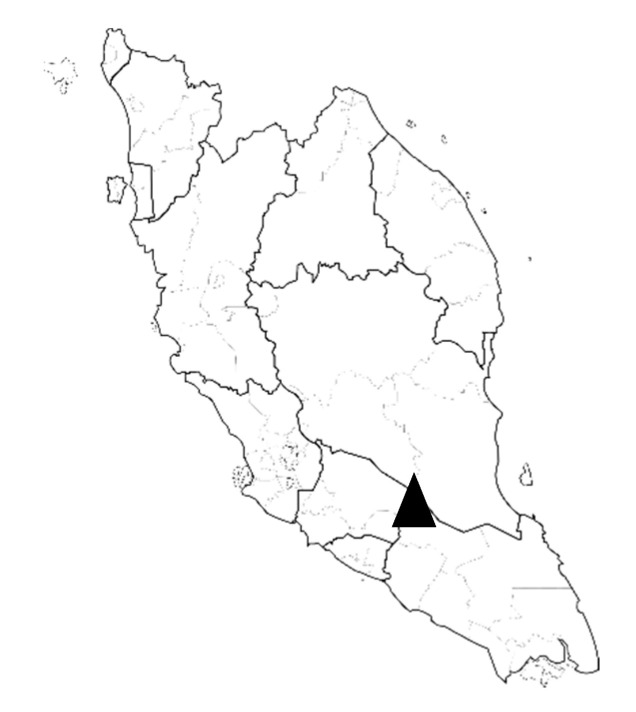
The location of honey sample collection in peninsular Malaysia.

Raw honey samples (*n* = 46) were harvested from jungles and secondary forests of Southern Negeri Sembilan, Northern Johor and South Western Pahang in peninsular Malaysia (Figure 2). Honey bee (*n* = 23) and stingless bee (*n* = 23) honey samples were collected from August 2016 to September 2018 (Table 6). The honey samples were manually filtered and bottled without heat treatment. All samples were kept in room temperature (23–26 °C) prior to analysis.

### 4.2. Bacterial Samples

As listed in Table 7, reference strains of Gram-positive bacteria, *Staphylococcus aureus* (ATCC 25923 and ATCC 33591) and *Escherichia coli* (ATCC 25922 and ATCC 35218) provided by the Faculty of Science, UTAR were used for antibacterial evaluation of honey samples. Furthermore, in the investigation of interactive effect between honey and antibiotics, four identified clinical isolates of *E. coli* were used. These isolates were obtained from a private hospital located in Penang, Malaysia. *S. aureus* and *E. coli* were cultured and maintained on mannitol salt agar and MacConkey agar, respectively.

### 4.3. Antibacterial Properties

#### 4.3.1. Agar-Well Diffusion Method

The inhibitory effect of each honey sample was evaluated based on a modified agar-well diffusion method [2,25]. Fresh overnight bacterial culture of *S. aureus* (ATCC 25923 and ATCC 33591) and *E. coli* (ATCC 25922 and ATCC 35218) was inoculated with 8 mL of sterile 0.85% normal saline. The turbidity of each bacterial suspension was adjusted to 0.5 McFarland (optical density reading 0.08–0.13 at the wavelength of 625 nm, which was equivalent to 1 × 10^8^ CFU/mL. The tip of a cotton swab was soaked into the bacterial suspension and pressed firmly to remove the excess fluid. Then, the bacterial suspension was streaked over the surface of agar evenly. A sterile 6-mm diameter cork borer was used to make wells on agar. Approximately 90 µL of honey sample was filled into one well, the same volume of distilled water which served as a negative control was filled into another well and ampicillin solution (10 µg/mL) which served as the positive control was added into a different well. After the inoculation of samples, the agar plates were incubated at 37 °C overnight (16–20 h). The diameter of the zone of inhibition (if any) was measured to the nearest centimeter (cm).

#### 4.3.2. Endotoxin Quantification

The bactericidal effect of honey samples against *E. coli* was determined by measuring the level of endotoxin utilizing Limulus Amebocyte Lysate (LAL) (Lonza, Walkersville, MD, USA). First, the 0-h sample was prepared by mixing 1800 µL of honey with 200 µL of 0.5 McFarland bacterial suspension. Then, the 24-h sample was prepared by incubating 1 mL of the prepared mixture at 37 °C for 24 h. Each 0-h and 24-h sample was adjusted to a pH range of 6.0–8.0 using sodium hydroxide (0.1 N) and hydrochloric acid (0.1 N) prior assay. Next, each 50 µL of sample or standard (0.0.125–1.0 EU/mL) was dispensed into an endotoxin-free reaction tube. A blank was prepared with the same volume of LAL reagent water. At time T = 0, 50 µL of LAL was added to each reaction tube, after 10 min, 100 µL of substrate solution which had been prewarmed to 37 °C was added. At T = 16 min, acetic acid was added to stop the reaction. Absorbance was read at 410 nm. Each sample was assessed in triplicate and the average value was calculated. The endotoxin level is expressed as endotoxin units per milliliter (EU/mL).

#### 4.3.3. Scanning Electron Microscopy

Prior processing, 0.50 mL of 0.5 McFarland *E. coli* suspension was incubated with 4.50 mL of honey at 37 °C for 24 h. The sample was then centrifuged at 3500 rpm for 5 min, the pellet was fixed with 2.5% (*v*/*v*) glutaraldehyde in 0.01 M phosphate buffer solution (PBS) for overnight. The sample was washed thrice for 10 min with 0.01 M PBS and subsequently by distilled water for another 10 min. The sample was dehydrated with ascending concentrations of ethanol solution, started with 25% (*v*/*v*) ethanol solution for 5 min followed by 50% (*v*/*v*) ethanol solution for 10 min, 75% (*v*/*v*) ethanol solution for 10 min, 95% (*v*/*v*) ethanol solution for 10 min and lastly with absolute ethanol for 10 min. After dehydration, the sample was subjected to freeze drying for 24 h. Thereafter, the sample was transferred to a carbon tape on copper stage and coated with platinum and viewed under JEOL JSM-6701F scanning electron microscope. The steps were repeated for negative control by replacing honey with normal saline added to the bacterial suspension.

#### 4.3.4. Determination of Antibacterial Factors

In order to determine the physicochemical properties that are mainly involved in antibacterial effects of honey, four solution samples including sugar solution (43% fructose, 28% glucose and 2.0% sucrose, g/100 g) [47], hydrogen peroxide solution (184 µmol/L), hydrochloric acid solution (pH 3.3) and gallic acid solution (104 mg GAE/kg) were formulated based on the physicochemical properties of honeydew honey produced by *H. itama* (unpublished data). The inhibitory and bactericidal effects of these samples were also assessed with agar-well diffusion method and endotoxin assay.

#### 4.3.5. Interactive Effect with Antibiotics

A modified agar-well diffusion method was performed to assess the interactive effect between honey and antibiotics [25]. Each *E. coli* suspension with 0.5 McFarland was prepared as stated earlier. A cotton swab was used to streak the prepared bacterial suspension evenly over the surface of agar. Wells with diameter of 0.6 cm were cut on the surface of agar by using a sterile cork borer. Each well was inoculated with 90 µL of honey (50%, *v*/*v*), ampicillin (32 µg/mL), mixture of honey (50%, *v*/*v*) and ampicillin (32 µg/mL), gentamicin (8 µg/mL), and a mixture of honey (50%, *v*/*v*) and gentamicin (8 µg/mL). Distilled water which served as the negative control was also inoculated in a different well. The agar plates were incubated at 37 °C overnight (16–20 h). After incubation, the diameter of zone of inhibition (if any) was measured to the nearest centimeter (cm).

#### 4.3.6. Statistical Analysis

Each assay was carried out in triplicates and conducted at room temperature (23–26 °C) unless stated otherwise. The data were expressed as means ± standard deviation. An independent t-test was performed using Microsoft Excel Analyse-it Standard Edition v5.50 to determine the significance of mean value differences at the level of significance of 0.05.

## 5. Conclusions

Generally, stingless bee honeydew honey exhibited greater antibacterial properties with both inhibitory and bactericidal effects. A synergistic effect was observed between this honey with antibiotics in inhibiting antibiotic resistant bacteria. Outcomes of this study reveal the potential of honeydew honey produced by the stingless bee *H. itama* which can serve as an antibacterial agent in health care.

## Figures and Tables

**Table 1:** The zone of inhibition (cm) of honey bee (A) and stingless bee (S) honey samples (100% *v*/*v*) against pathogenic bacteria.

Sample	*S. aureus* (ATCC 25923) *	*S. aureus* (ATCC 33591)	*E. coli* (ATCC 25922) *^	*E. coli* (ATCC 35218) *^
A1	Nil	Nil	1.0 ± 0.2	0.9 ± 0
A2	Nil	Nil	0.9 ± 0.1	0.9 ± 0
A3	Nil	Nil	0.8 ± 0.1	0.8 ± 0
A4	0.8 ± 0	Nil	0.9 ± 0	0.8 ± 0
A5	0.9 ± 0.1	0.9 ± 0	0.9 ± 0	0.8 ± 0
A6	0.8 ± 0.1	0.8 ± 0	0.9 ± 0.1	0.8 ± 0
A7	Nil	Nil	1.0 ± 0.2	0.8 ± 0
A8	Nil	Nil	0.9 ± 0.1	0.8 ± 0.1
A9	Nil	Nil	0.7 ± 0.1	0.7 ± 0.1
A10	Nil	Nil	0.7 ± 0	0.7 ± 0
A11	Nil	Nil	0.9 ± 0.1	0.8 ± 0.1
A12	Nil	Nil	1.0 ± 0.1	0.8 ± 0.1
A13	Nil	Nil	Nil	Nil
A14	Nil	Nil	Nil	Nil
A15	Nil	Nil	Nil	0.8 ± 0.1
A16	Nil	Nil	0.8 ± 0.1	Nil
A17	Nil	Nil	0.8 ± 0.1	Nil
A18	Nil	Nil	0.8 ± 0	Nil
A19	Nil	Nil	0.7 ± 0	Nil
A20	Nil	Nil	0.8 ± 0	Nil
A21	0.9 ± 0.1	0.8 ± 0	1.0 ± 0	0.8 ± 0.1
A22	0.8 ± 0.1	Nil	1.1 ± 0.1	0.8 ± 0.1
A23	0.8 ± 0.1	0.8 ± 0	0.9 ± 0.1	0.7 ± 0
Average	0.8 ± 0.1	0.8 ± 0.1	0.9 ± 0.1	0.8 ± 0.1
*A. cerana* honeydew (A1–A9)	0.8 ± 0.1	0.9 ± 0.1	0.9 ± 0.1	0.8 ± 0.1
*A. cerana* blossom (A10–A23)	0.8 ± 0.1	0.8 ± 0	0.9 ± 0.1	0.8 ± 0
S1	1.2 ± 0.1	1.0 ± 0.1	1.5 ± 0.1	1.4 ± 0.1
S2	1.1 ± 0	0.9 ± 0.2	1.4 ± 0.1	1.3 ± 0.1
S3	1.1 ± 0	0.9 ± 0	1.6 ± 0	1.3 ± 0.1
S4	1.4 ± 0.1	0.9 ± 0.1	1.7 ± 0.1	1.5 ± 0
S5	1.0 ± 0.1	0.9 ± 0	1.1 ± 0.1	1.2 ± 0
S6	0.9 ± 0.1	0.9 ± 0.1	0.9 ± 0.1	1.0 ± 0
S7	0.9 ± 0.1	0.8 ± 0	1.2 ± 0.1	1.2 ± 0
S8	0.9 ± 0.1	0.8 ± 0	1.0 ± 0	1.1 ± 0
S9	0.7 ± 0.1	0.8 ± 0.1	1.1 ± 0	0.9 ± 0
S10	0.8 ± 0.1	0.8 ± 0	1.0 ± 0.1	0.9 ± 0.1
S11	0.9 ± 0.1	0.8 ± 0	1.1 ± 0.1	0.9 ± 0
S12	0.9 ± 0.1	0.7 ± 0	0.9 ± 0.1	0.7 ± 0
S13	0.8 ± 0.1	0.7 ± 0	1.0 ± 0.1	0.7 ± 0.1
S14	0.8 ± 0.1	0.8 ± 0	1.0 ± 0.1	0.7 ± 0.2
S15	0.8 ± 0.1	0.8 ± 0.1	1.0 ± 0.1	0.8 ± 0.1
S16	0.8 ± 0.1	0.8 ± 0	0.9 ± 0.1	0.7 ± 0
S17	1.0 ± 0.1	0.8 ± 0.1	1.3 ± 0.1	1.0 ± 0
S18	1.0 ± 0.1	0.8 ± 0	1.3 ± 0.1	1.0 ± 0.1
S19	1.2 ± 0.1	0.9 ± 0	1.3 ± 0.1	1.0 ± 0
S20	0.9 ± 0	0.9 ± 0	1.1 ± 0.1	0.8 ± 0.1
S21	1.0 ± 0.1	0.9 ± 0.1	1.0 ± 0	0.8 ± 0.1
S22	1.0 ± 0.1	0.8 ± 0	1.3 ± 0.1	1.0 ± 0.1
S23	1.0 ± 0.1	0.9 ± 0.1	1.2 ± 0.1	1.1 ± 0
Average	1.0 ± 0.2	0.8 ± 0.1	1.2 ± 0.2	1.0 ± 0.2
*H. itama* honeydew (S1–S8)	1.1 ± 0.2	0.9 ± 0.1	1.3 ± 0.3	1.3 ± 0.2
*H. itama* blossom (S9–S16)	0.8 ± 0.1 ^a^	0.8 ± 0 ^a^	1.0 ± 0.1 ^a^	0.8 ± 0.1 ^a^
*G. thoracica* blossom (S17–S23)	1.0 ± 0.1	0.9 ± 0.1	1.2 ± 0.1	1.0 ± 0.1 ^b^

Nil—No zone of inhibition. *—Significant different between honey bee honey and stingless bee honey at *p* < 0.05. ^—The zone of inhibition exhibited on *E. coli* was significantly larger than *S. aureus* at *p* < 0.05. ^a^—Significant different with *H. itama* honeydew honey at *p* < 0.05. ^b^—Significant different with *H. itama* honeydew honey at *p* < 0.05.

**Table 2:** The endotoxin level (EU/mL) released by *E. coli* treated with stingless bee honey samples (100% *v*/*v*) after 0-h and 24-h incubation.

	*E. coli* (ATCC 25922)		*E. coli* (ATCC 35218)	
Sample	0-h	24-h ^	0-h	24-h ^
S1	1.77 ± 0	2.32 ± 0	1.68 ± 0	2.32 ± 0
S2	1.58 ± 0	2.22 ± 0	1.51 ± 0	2.21 ± 0
S3	1.88 ± 0	2.30 ± 0	1.58 ± 0	2.20 ± 0
S4	1.56 ± 0	2.21 ± 0	1.56 ± 0	2.21 ± 0
S5	1.72 ± 0	2.29 ± 0	1.62 ± 0	2.25 ± 0
S6	1.89 ± 0	2.35 ± 0	1.69 ± 0	2.30 ± 0
S7	1.97 ± 0	2.38 ± 0	1.67 ± 0	2.30 ± 0
S8	1.88 ± 0	2.32 ± 0	1.88 ± 0	2.32 ± 0
S9	1.33 ± 0	2.12 ± 0	1.31 ± 0	2.10 ± 0
S10	1.38 ± 0	2.12 ± 0	1.35 ± 0	2.11 ± 0
S11	1.27 ± 0	2.02 ± 0	1.37 ± 0	2.09 ± 0
S12	1.38 ± 0	2.05 ± 0	1.38 ± 0	2.05 ± 0
S13	1.27 ± 0	2.00 ± 0	1.25 ± 0	2.00 ± 0
S14	1.38 ± 0	2.15 ± 0	1.30 ± 0	2.05 ± 0
S15	1.37 ± 0	2.13 ± 0	1.35 ± 0	2.10 ± 0
S16	1.38 ± 0	2.12 ± 0	1.33 ± 0	2.12 ± 0
S17	1.67 ± 0	2.22 ± 0	1.56 ± 0	2.20 ± 0
S18	1.68 ± 0	2.31 ± 0	1.58 ± 0	2.22 ± 0
S19	1.57 ± 0	2.22 ± 0	1.50 ± 0	2.23 ± 0
S20	1.57 ± 0	2.19 ± 0	1.57 ± 0	2.18 ± 0
S21	1.57 ± 0	2.17 ± 0	1.57 ± 0	2.16 ± 0
S22	1.50 ± 0	2.15 ± 0	1.48 ± 0	2.14 ± 0
S23	1.57 ± 0	2.19 ± 0	1.58 ± 0	2.29 ± 0
Average	1.57 ± 0.21	2.20 ± 0.10	1.51 ± 0.16	2.18 ± 0.09
*H. itama* honeydew (S1–S8)	1.78 ± 0.15 *	2.30 ± 0.06 *	1.65 ± 0.11 *	2.26 ± 0.05 *
*H. itama* blossom (S9–S16)	1.35 ± 0.05	2.09 ± 0.06	1.33 ± 0.04	2.08 ± 0.04
*G. thoracica* blossom (S17–S23)	1.59 ± 0.06	2.21 ± 0.05	1.55 ± 0.04	2.20 ± 0.05

^—Significant different between 0-h and 24-h at *p* < 0.05. *—Significant different with *H. itama* blossom honey and *G. thoracica* blossom honey at *p* < 0.05.

**Table 3:** The zone of inhibition (cm) and endotoxin level (EU/mL) released by *E. coli* treated with different solutions.

Sample	Zone of Inhibition		Endotoxin Level			
	*E. coli* (ATCC 25922)	*E. coli* (ATCC 35218)	*E. coli* (ATCC 25922)		*E. coli* (ATCC 35218)	
			0-h	24-h	0-h	24-h
Sugar solution	Nil	Nil	1.20 ± 0	1.25 ± 0	1.07 ± 0	1.10 ± 0
Hydrogen peroxide solution	Nil	Nil	1.41 ± 0	1.52 ± 0	1.22 ± 0	1.35 ± 0
Acid solution	Nil	Nil	1.54 ± 0	1.58 ± 0	1.29 ± 0	1.34 ± 0
Gallic acid solution	Nil	Nil	1.22 ± 0	1.23 ± 0	1.11 ± 0	1.10 ± 0

Nil—No zone of inhibition.

**Table 4:** Antibiotic susceptibility profile of each *E. coli* clinical isolate.

Antibiotic	*E. coli* 1	*E. coli* 2	*E. coli* 3	*E. coli* 4
Ampicillin (10 µg)	R	R	R	R
Chloramphenicol (30 µg)	S	S	R	S
Gentamicin (10 µg)	S	S	S	S
Tetracycline (30 µg)	S	R	R	R

R—Resistant; S—Susceptible.

**Table 5:** Antibacterial activity of *H. itama* honeydew honey (50% *v*/*v*), ampicillin (32 µg/mL), gentamicin (8 µg/mL) separately and combined against *E. coli* isolates.

Sample	*E. coli* 1	*E. coli* 2	*E. coli* 3	*E. coli* 4	*E. coli* (ATCC 25922)	*E. coli* (ATCC 35218)
Honey	0.7 ± 0.1	0.7 ± 0	1.0 ± 0.1	0.7 ± 0.1	1.2 ± 0	1.0 ± 0
Ampicillin	Nil	0.7 ± 0.1	Nil	Nil	1.0 ± 0.1	Nil
Honey + Ampicillin	0.9 ± 0 (S)	1.3 ± 0 (S)	1.4 ± 0.1 (S)	0.7 ± 0.1	1.7 ± 0 (S)	1.0 ± 0.1
Gentamicin	1.3 ± 0	1.0 ± 0	Nil	1.3 ± 0	2.0 ± 0	1.6 ± 0
Honey + Gentamicin	1.3 ± 0.1	1.3 ± 0.1 (S)	1.4 ± 0 (S)	0.9 ± 0.1	2.2 ± 0 (S)	1.3 ± 0.1

Nil—No zone of inhibition. (S)—Synergistic effect achieved.

**Table 6:** Bee type and origin information of honey samples.

Sample	Bee Type	Bee Species	Origin	Collection
A1	Honey bee	*Apis cerana*	Honeydew	November 2016
A2	Honey bee	*Apis cerana*	Honeydew	May 2017
A3	Honey bee	*Apis cerana*	Honeydew	June 2017
A4	Honey bee	*Apis cerana*	Honeydew	April 2018
A5	Honey bee	*Apis cerana*	Honeydew	July 2018
A6	Honey bee	*Apis cerana*	Honeydew	September 2018
A7	Honey bee	*Apis cerana*	Honeydew	November 2016
A8	Honey bee	*Apis cerana*	Honeydew	April 2017
A9	Honey bee	*Apis cerana*	Honeydew	June 2017
A10	Honey bee	*Apis cerana*	Blossom	October 2016
A11	Honey bee	*Apis cerana*	Blossom	May 2017
A12	Honey bee	*Apis cerana*	Blossom	July 2017
A13	Honey bee	*Apis cerana*	Blossom	March 2018
A14	Honey bee	*Apis cerana*	Blossom	June 2018
A15	Honey bee	*Apis cerana*	Blossom	October 2018
A16	Honey bee	*Apis cerana*	Blossom	November 2016
A17	Honey bee	*Apis cerana*	Blossom	April 2017
A18	Honey bee	*Apis cerana*	Blossom	July 2017
A19	Honey bee	*Apis cerana*	Blossom	November 2016
A20	Honey bee	*Apis cerana*	Blossom	April 2017
A21	Honey bee	*Apis cerana*	Blossom	March 2018
A22	Honey bee	*Apis cerana*	Blossom	June 2018
A23	Honey bee	*Apis cerana*	Blossom	October 2018
S1	Stingless bee	*Heterotrigona itama*	Honeydew	August 2016
S2	Stingless bee	*Heterotrigona itama*	Honeydew	November 2016
S3	Stingless bee	*Heterotrigona itama*	Honeydew	April 2017
S4	Stingless bee	*Heterotrigona itama*	Honeydew	July 2017
S5	Stingless bee	*Heterotrigona itama*	Honeydew	September 2017
S6	Stingless bee	*Heterotrigona itama*	Honeydew	April 2018
S7	Stingless bee	*Heterotrigona itama*	Honeydew	July 2018
S8	Stingless bee	*Heterotrigona itama*	Honeydew	September 2018
S9	Stingless bee	*Heterotrigona itama*	Blossom	August 2016
S10	Stingless bee	*Heterotrigona itama*	Blossom	November 2016
S11	Stingless bee	*Heterotrigona itama*	Blossom	May 2017
S12	Stingless bee	*Heterotrigona itama*	Blossom	July 2017
S13	Stingless bee	*Heterotrigona itama*	Blossom	September 2017
S14	Stingless bee	*Heterotrigona itama*	Blossom	April 2018
S15	Stingless bee	*Heterotrigona itama*	Blossom	May 2018
S16	Stingless bee	*Heterotrigona itama*	Blossom	July 2018
S17	Stingless bee	*Geniotrigona thoracica*	Blossom	October 2016
S18	Stingless bee	*Geniotrigona thoracica*	Blossom	December 2016
S19	Stingless bee	*Geniotrigona thoracica*	Blossom	April 2017
S20	Stingless bee	*Geniotrigona thoracica*	Blossom	July 2017
S21	Stingless bee	*Geniotrigona thoracica*	Blossom	March 2018
S22	Stingless bee	*Geniotrigona thoracica*	Blossom	June 2018
S23	Stingless bee	*Geniotrigona thoracica*	Blossom	October 2018

A—Honey bee honey. S—Stingless bee honey.

**Table 7:** Bacteria samples used.

Bacteria Sample	Origin of Isolate
*S. aureus*	Reference strain, ATCC 25923
*S. aureus*	Reference strain, ATCC 33591
*E. coli*	Reference strain, ATCC 25922
*E. coli*	Reference strain, ATCC 35218
*E. coli* 1	Clinical strain isolated from urine sample
*E. coli* 2	Clinical strain isolated from urine sample
*E. coli* 3	Clinical strain isolated from urine sample
*E. coli* 4	Clinical strain isolated from ascitic fluid
